# Comparative Analysis of Progression Milestones Across Parkinson’s Disease Clinical, Pathological, and Data-driven Subtypes: A 10-year follow-up

**DOI:** 10.21203/rs.3.rs-6574563/v1

**Published:** 2025-06-23

**Authors:** Ahmed Negida, Nitai Mukhopadhyay, Brian D. Berman, Matthew J. Barrett

**Affiliations:** Virginia Commonwealth University

**Keywords:** Parkinson’s Disease, Subtyping, Disease Progression, Disease-Modification

## Abstract

Parkinson’s disease (PD) heterogeneity complicates disease-modification clinical trial design and lead to ambiguous results, necessitating robust subtyping frameworks to identify rapid progressors. This study compared progression milestones across previously defined clinical (tremor-dominant [TD], postural instability/gait difficulty [PIGD], indeterminate), pathological (brain-first, body-first), and data-driven (diffuse malignant [DM], intermediate [IM], mild motor-predominant [MMP]) PD subtypes. Using data from the Parkinson’s Progression Markers Initiative (PPMI), we analyzed milestone attainment across six functional domains and performed competing risks regression. Randomized controlled trial sample size simulations evaluated the impact of subtyping on trial efficiency. Data-driven subtypes exhibited the highest progression rates, with DM patients attaining 50% of milestones, surpassing PIGD (43.0%) and body-first (42.0%) subtypes. Competing risks regression confirmed that DM patients had more than double the hazard of progression compared to MMP (SHR 2.02, 95% CI 1.49 to 2.75; P < 0.001). Trial power simulations demonstrated that enrolling DM patients could reduce sample size requirements by approximately 50% using standard trial durations compared to unstratified PD cohorts. This analysis showed that data-driven subtyping, particularly the identification of the DM subtype, offers a promising strategy to optimize disease-modification trials in PD by capturing patients who meet progression milestones earlier.

## INTRODUCTION

Parkinson’s disease (PD) is a progressive neurodegenerative disorder affecting selectively vulnerable neuronal populations including dopaminergic neurons in the substantia nigra pars compacta, cholinergic neurons in the pedunculopontine nucleus, and noradrenergic neurons in the locus coeruleus [1, 2]. The cardinal pathological feature of PD is the accumulation of misfolded α-synuclein aggregates, known as Lewy bodies [3–6]. The classical motor triad—bradykinesia, resting tremor, and rigidity—often dominates early clinical presentation, later progressing to postural instability and gait disturbances. Non-motor manifestations of PD include rapid eye movement (REM) sleep behavior disorder (RBD), autonomic dysfunction, hyposmia, and psychiatric symptoms, some of which may precede motor symptoms by decades [7–9]. Clinically, PD can show marked clinical heterogeneity in terms of the motor and non-motor manifestations as well as the disease progression trajectories.

Current therapies, primarily focused on dopamine replacement (e.g., levodopa), provide symptomatic relief for motor symptoms but fail to modify disease progression [10, 11]. Currently, there is no available pharmacological therapies that can stop or slow the disease progression, despite the several disease-modification trials that have been conducted over the last three decades. Two major challenges are believed to contribute to the failure of disease-modifying trials in PD to date: (1) substantial heterogeneity in pathology, symptoms, and progression trajectories, and (2) the slow progression of the disease in most patients, contrasted with rapid deterioration in others. This variability poses a challenge for conventional trial designs, which typically rely on short-term follow-up periods (1–2 years) insufficient to capture clinically meaningful progression in many patients.

To address the heterogeneity seen in PD, efforts have been made to stratify PD patients into biologically or clinically homogeneous subtypes [12–16]. The most widely described PD subtypes include (1) clinical classifications, such as tremor-dominant (TD) and postural instability/gait difficulty (PIGD) [17, 18]; (2) pathological models, including brain-first and body-first subtypes, which hypothesize distinct etiological pathways [16, 19]; and (3) data-driven categories, such as the diffuse malignant (DM), intermediate (IM), and mild motor-predominant (MMP) subtypes, identified through machine learning-based cluster analysis [14, 15].

Despite these efforts, critical limitations hinder the translation of subtyping research into improved clinical trial design. The Movement Disorders Society Task Force on the definition of PD stated that “*subtypes should only be delineated if there are clear data that demonstrate consistent, large differences in prognosis, predicted disease manifestations, or treatment.*” [20] and the literature emphasized that the key criteria for PD subtyping are biomarker profile and longitudinal progression [14, 15]. Existing subtyping models have not been directly compared for their prognostic accuracy and most studies rely on cross-sectional data without demonstrating, through longitudinal study, the prognostic value of these subtypes in future clinical trials of potential disease modifying interventions. This knowledge gap limits the applicability of PD subtypes in optimizing disease-modification clinical trials, where enrolling rapid progressors is important to detect treatment effects efficiently. Our study addresses this gap by answering an important research question “*Which existing PD subtype classification—clinical, pathological, or data-driven—most effectively identifies individuals with the fastest progression across six key domains: motor complications, gait/balance, cognition, autonomic dysfunction, functional dependence, and activities of daily living?*”. Using longitudinal data from the Michael J. Fox Foundation’s Parkinson’s Progression Markers Initiative (PPMI) cohort, we tracked 25 disease progression milestones for each of the 3 subtyping models and simulated clinical trials to assess the utility of each subtyping model. The goal of this work was to identify subtypes that enhance trial sensitivity to detect treatment effects, thereby accelerating therapeutic development.

## METHODS

### Study Design

This study used data from the PPMI, a prospective, longitudinal, observational cohort study designed to identify biomarkers of PD progression. Ethical approval was obtained from institutional review boards at all participating PPMI sites, and written informed consent was provided by all participants. The current analysis was exempt from additional ethical review at Virginia Commonwealth University (VCU) as it involved secondary analysis of de-identified data. Reporting adhered to the STROBE (Strengthening the Reporting of Observational Studies in Epidemiology) guidelines for observational studies.

### Study Participants

PPMI PD participants inclusion criteria were as follows: (1) diagnosis within the past two years, (2) Hoehn and Yahr stage I–II, and (3) presence of bradykinesia plus at least one additional motor symptom (resting tremor or rigidity). Individuals with a Montreal Cognitive Assessment (MoCA) score < 21 were excluded. Data were extracted from the PPMI database on September 28, 2024, encompassing all eligible participants with a cohort definition of “*Parkinson’s Disease*”. We excluded patients in the Scans Without Evidence of Dopaminergic Deficit (SWEDD) cohort and prodromal cohort.

### Study Assessments

Participants underwent standardized clinical and neuropsychological assessments, including global cognition measured by the MoCA, motor function evaluated using the Movement Disorder Society–Unified Parkinson’s Disease Rating Scale (MDS-UPDRS Parts I–III), autonomic symptoms assessed by the Scale for Outcomes in Parkinson’s Disease–Autonomic (SCOPA-AUT), olfaction measured by the University of Pennsylvania Smell Identification Test (UPSIT), and REM sleep behavior assessed using the REM Sleep Behavior Disorder Screening Questionnaire (RBDSQ).

Cerebrospinal fluid (CSF) biomarkers analyzed in this study included amyloid-beta 1–42 (Aβ_42_), total tau (tTau), phosphorylated tau at threonine 181 (pTau), and neurofilament light chain (NfL). Serum NfL levels were also measured. To align with the subtyping time point, only biomarker measurements obtained at the baseline visit were included. Aβ_42_, tTau, and pTau were quantified using Elecsys immunoassays (Roche), while CSF and serum NfL concentrations were measured using the Simoa platform (Quanterix).

### Subtyping Classifications

Three distinct subtyping frameworks were applied at baseline:

1. Clinical Subtypes
  Participants were classified into TD, PIGD, or indeterminate categories based on the MDS-UPDRS following the recommended criteria [18]. The tremor score was derived from the sum of items 2.10 (tremor presence) and 3.15–3.18 (resting tremor severity in limbs), while the PIGD score was calculated from items 2.12–2.13 (gait and postural instability) and 3.10–3.12 (axial motor features). Ratios of tremor to PIGD scores were computed, with thresholds of ≥ 1.15 defining TD, ≤ 0.9 defining PIGD, and values between 0.9–1.15 categorized as indeterminate.
2. Pathological Subtypes
  Brain-first and body-first subtypes were defined using the RBDSQ as described before [19]. Participants with RBDSQ scores ≤ 3, indicating minimal REM sleep dysfunction, were classified as brain-first. Those with scores ≥ 6, reflecting prominent REM sleep features, were classified as body-first. Individuals scoring 4–5 were excluded to ensure clear distinction between subtypes.
3. Data-Driven Subtypes: Subtypes (DM, IM, and MMP) were assigned using criteria established by Fereshtehnejad *et al*.[14]. Composite motor scores (sum of MDS-UPDRS Parts II and III) and non-motor scores (SCOPA-AUT for autonomic dysfunction, RBDSQ for REM sleep behavior disorder, and MoCA for cognition) were computed. Using data of the full cohort (n = 1220 PD patients), we calculated the 75th percentiles for all motor and non-motor scores; for the MoCA, we calculated the 25th percentile to adjust for the different score direction of MoCA. Then we applied the subtyping criteria as detailed before [14]: DM required motor scores > 75th percentile plus ≥ 1 non-motor score > 75th percentile or all 3 non-motor scores > 75th percentile; MMP required motor scores < 75th percentile with all non-motor scores < 75th percentile; IM included all other participants.

### Progression Milestones Tracking

Progression milestones were assessed longitudinally across all available follow-up visits in the PPMI cohort as described before [21]. A total of 25 clinically meaningful milestones were defined across six domains: walking and balance, motor complications, cognition, autonomic dysfunction, functional dependence, and activities of daily living. Milestones were evaluated during all available scheduled visits up to 10 years. Participants were considered milestone-positive from the first visit at which criteria for any milestone were met. Once a milestone was attained, participants were classified as having reached that milestone, and progression rates were analyzed both overall and by domain. Missing data were conservatively handled by assuming that milestone criteria were not met when assessments were unavailable.

### Study Size and Power Calculation

This study analyzed all eligible participants from the PPMI cohort, yielding a total sample size of 1,220 patients. No formal priori sample size calculation was performed given the secondary nature of the analysis. However, post-hoc estimations indicated that the available sample provided sufficient statistical power. For comparisons of milestone attainment rates across subtypes, the study had > 80% power to detect absolute differences of 10–15% between groups at a two-tailed significance level of 0.05, corresponding to a medium-to-large effect size (Cohen’s h ≈ 0.4–0.6). In competing risks regression analyses, the sample size provided > 80% power to detect subdistribution hazard ratios (SHRs) of 1.5 or greater, assuming an event rate of approximately 30% and an α = 0.05. Given observed SHR estimates ranging from 1.36 to 2.06 in key comparisons, and the substantial number of events recorded, the study was adequately powered to detect clinically meaningful differences across subtypes.

### Statistical Analysis

Continuous variables were summarized as median and interquartile range (IQR), while categorical variables were presented as frequencies and proportions. For comparisons between two groups (pathological subtypes: brain-first vs. body-first), the Wilcoxon rank-sum test was used. For comparisons across three groups (clinical subtypes: TD, PIGD, indeterminate; and data-driven subtypes: DM, IM, MMP), the Kruskal-Wallis test was applied for continuous variables. Pearson chi-square tests were used to assess differences in categorical variables. Competing risks regression models, based on the Fine-Gray method, were employed to estimate SHRs for milestone attainment across subtypes, adjusting for age at enrollment. Statistical significance was set at a two-tailed P-value < 0.05. All analyses were conducted using Jamovi version 2.3.28 (The Jamovi Project), Stata/MP 17.0 (StataCorp, College Station, TX, USA), and R version 4.5.0 (R Foundation for Statistical Computing, Vienna, Austria) within the RStudio integrated development environment (version 2024.12.1 + 563; Posit Software, PBC, 2025).

### Hypothesized Disease-Modifying Trial Sample Size Estimations

We estimated the minimum sample sizes required for disease-modifying trials in Parkinson’s disease using observed survival probabilities and a hypothesized treatment effect. Patients who had reached any progression milestone at baseline were excluded to ensure that only incident milestones were considered. Time-to-event data were structured using Stata (*stset*), with event status coded as failure. Survival probabilities at 1–10 years were extracted for each subtype using the Kaplan-Meier method (*sts list by subtype*). Progression event probabilities were calculated as (1 – survival probability). Assuming a 20% relative reduction in milestone progression (HR = 0.80), we applied standard formulas for time-to-event sample size estimation with 80% power and a two-sided alpha of 0.05. This effect size (20% efficacy) is supported by a recent modeling study by Ribba *et al*., which simulated PD progression and evaluated treatment effects ranging from 20–50% slowing of decline using longitudinal data from PPMI and PASADENA trial [22]. All estimations were implemented in Python 3.10 (Google Colab), using survival probabilities derived from Stata/MP 17.0.

## RESULTS

### Clinical Characteristics of the Study Groups

A total of 1,220 patients from the PPMI cohort were included. Table 1 summarizes the baseline characteristics across clinical, pathological, and data-driven subtypes. Age at enrollment, age at symptom onset, and age at diagnosis did not significantly differ across clinical (TD, PIGD, indeterminate) or pathological (brain-first, body-first) subtypes. However, data-driven subtypes showed significant differences in age at enrollment and diagnosis (P < 0.01), with DM patients being older than MMP and IM subtypes. Male sex distribution was comparable across all groups.

Motor severity differed across all subtype classifications. PIGD and Indeterminate clinical subtypes exhibited higher baseline MDS-UPDRS II and III scores compared to TD (P < 0.01). Similarly, body-first patients had higher MDS-UPDRS scores than brain-first patients (P < 0.01). Among data-driven subtypes, DM patients demonstrated the highest baseline motor burden (MDS-UPDRS II: median 11.0, MDS-UPDRS III: median 33.5). Regarding cognitive and autonomic function, no significant differences in MoCA scores were observed among groups, while SCOPA-AUT scores were highest in DM patients (median = 15), reflecting greater baseline autonomic dysfunction (P < 0.01).

### Biomarkers in different PD subtypes

Significant differences in CSF phosphorylated tau (pTau) and total tau (tTau) concentrations were observed among clinical subtypes (*P* = 0.01 and *P* = 0.03, respectively), with PIGD patients exhibiting the highest median levels. No significant differences were found in CSF Aβ_42_ or NfL concentrations across clinical or pathological subtypes. However, data-driven subtypes showed significant differences in NfL levels (Table 2). In the post-hoc analysis, serum NfL concentrations were significantly elevated in the DM subtype compared to the MMP and IM subtypes (*P* < 0.01).

### Milestone Attainment Across Subtypes

**Figure 1:**
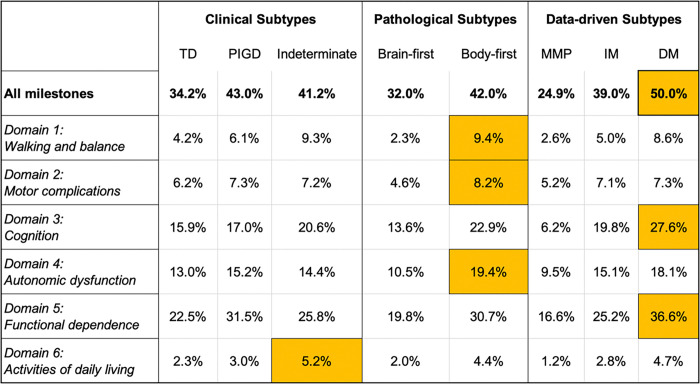
TD=Tremor dominant; PIGD=Postural Instability Gait Disorder; MMP=Mild Motor Predominant; IM=Intermediate; DM=Diffuse Malignant; Percentage of patients reaching progression milestones within 10 years across Parkinson’s disease subtypes, strati ed by clinical (TD, PIGD, Indeterminate), pathological (Brain-first, Body-first), and data-driven (MMP, IM, DM) subtypes. Bolded values represent the total milestone frequency within each subtype. Cells highlighted in orange indicate the highest proportion within each domain row.

Progression milestone attainment rates over 10 years varied substantially across clinical, pathological, and data-driven subtypes. Among clinical subtypes, the PIGD group showed the highest overall milestone attainment (43.0%), followed closely by the indeterminate subtype (41.2%), and then the TD group (34.2%). For pathological classifications, body-first patients reached more milestones (42.0%) than brain-first patients (32.0%). Notably, among data-driven subtypes, the DM group exhibited the highest rate of progression (50.0%), exceeding the IM (39.0%), the MMP (24.9%), and all other subtypes (Fig. 1).

**Figure 2:**
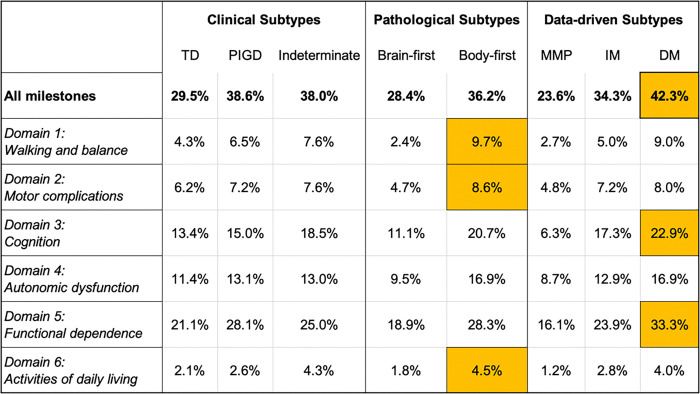
TD=Tremor dominant; PIGD=Postural Instability Gait Disorder; MMP=Mild Motor Predominant; IM=Intermediate; DM=Diffuse Malignant; Percentage of Parkinson’s disease patients reaching new progression milestones within 10 years—excluding individuals who met milestones at baseline—across clinical (TD, PIGD, Indeterminate), pathological (Brain-first, Body-first), and data-driven (MMP, IM, DM) subtypes. Bolded values reflect the overall milestone frequency in each subtype. Cells highlighted in orange indicate the highest percentage per domain across all subtype groups.

After excluding patients who had already reached milestones at baseline, similar trends persisted (Fig. 2). The DM subtype continued to show the highest incidence of new milestone acquisition (42.3%), followed by PIGD (38.6%) and body-first (36.2%) subtypes. Again, domain-specific patterns emphasized the aggressiveness of the DM subtype, with leading rates in cognition (22.9%) and functional dependence (33.3%).

### Survival Analysis of Disease Progression Milestones

**Figure 3:**
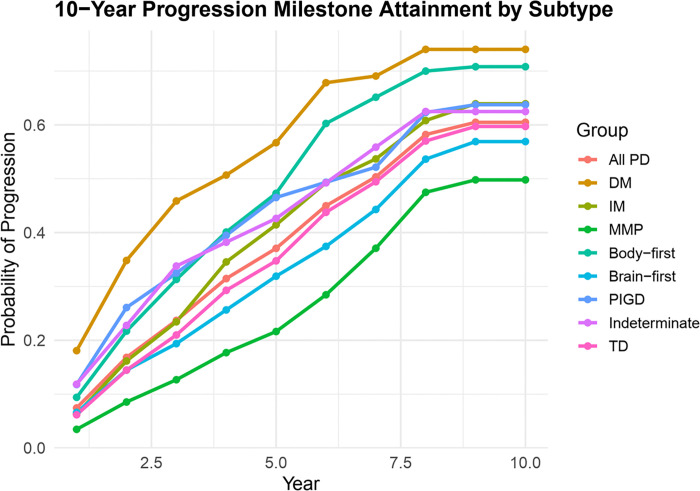
TD=Tremor dominant; PIGD=Postural Instability Gait Disorder; MMP=Mild Motor Predominant; IM=Intermediate; DM=Diffuse Malignant; Kaplan-Meier failure functions showing the cumulative probability of progression milestone attainment across Parkinson’s disease subtypes over 10 years. The DM subtype showed the highest cumulative risk (~70%), indicating the fastest disease progression. Y-axis represents the estimated probability of progression (1 – survival).

Progression risk over time differed markedly by subtype, as visualized in Fig. 3. The DM subtype demonstrated the steepest trajectory, reaching an estimated 70% cumulative progression probability by year 10 based on Kaplan-Meier failure functions (1 – survival), followed by the body-first group. In contrast, clinical subtypes such as tremor-dominant and MMP exhibited slower progression.

**Figure 4:**
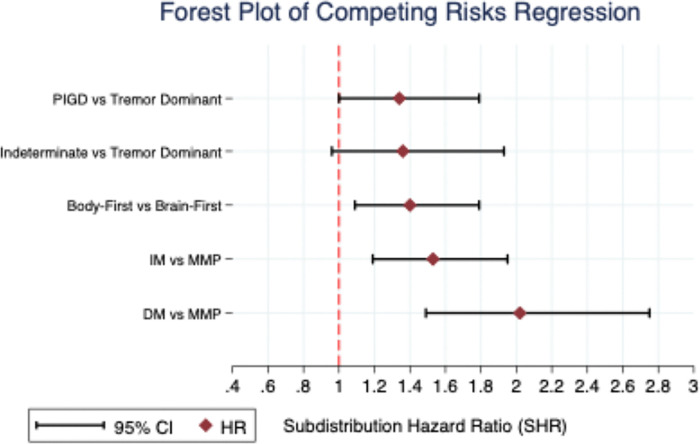
PIGD=Postural Instability Gait Disorder; MMP=Mild Motor Predominant; IM=Intermediate; DM=Diffuse Malignant; Forest plot summarizing SHR for progression milestone attainment across subtype comparisons; comparisons include clinical (PIGD vs. TD, and Indeterminate vs. TD), pathological (body-first vs. brain-first), and data-driven subtypes (DM vs. MMP, IM vs. MMP); red dashed line represents SHR=1.0 as the reference; hazard ratios are adjusted for age at enrollment; larger hazard ratios indicate faster progression rates.

These trends were further quantified using the Fine-Gray model for competing risks regression (Table 3, Fig. 4). Among pathological subtypes, body-first patients had a significantly higher hazard of milestone attainment than brain-first patients (SHR = 1.40; 95% CI, 1.09 to 1.79; P = 0.007). Within data-driven subtypes, DM patients progressed more than twice as fast as MMP patients (SHR = 2.02; 95% CI, 1.49 to 2.75; P < 0.001), and IM patients also had elevated risk (SHR = 1.53; 95% CI, 1.19 to 1.95; P = 0.001). In contrast, differences among clinical subtypes were smaller with marginal statistical significance (Table 3). Across all models, enrollment age had a modest effect on progression, while subtype classification remained the strongest independent predictor.

### Power-based Sample Size Estimations

**Figure 5:**
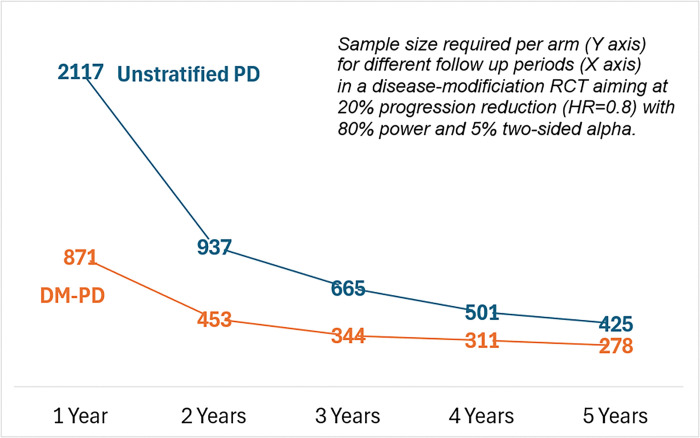
DM-PD=Diffuse Malignant Parkinson’s Disease; Estimated sample size and trial duration requirements for detecting a 20% reduction in progression milestones (HR=0.80) with 80% statistical power and a two-sided alpha of 0.05. The solid orange line represents the DM subtype cohort, while the blue line represents the unstratified PD cohort. Numbers indicate the minimum sample size per arm required to achieve 80% power for trial durations (1–5 years). DM subtype trials consistently required smaller sample sizes and shorter durations compared to trials enrolling unstratified PD patients.

We performed sample size estimations using 1 – Kaplan-Meier survival estimates to model cumulative event probabilities. We explored how patient strati cation influences trial design efficiency. Assuming a hypothetical disease-modifying therapy that reduces the hazard of reaching a new progression milestone by 20% (HR = 0.80), we estimated the minimum required sample sizes per trial arm to achieve 80% power at a two-sided alpha of 0.05. Event probabilities (1 – survival) were derived from time-to-event data for each subgroup. As illustrated in Fig. 5, trials targeting the DM subtype required substantially fewer participants and shorter follow-up durations to meet power thresholds compared to trials enrolling unstratified PD patients. For example, a 2-year trial would require only 453 participants per arm if limited to DM-PD patients, versus 937 per arm in the unstratified PD cohort.

## DISCUSSION

This study showed that data-driven subtyping, particularly the DM subtype, most effectively identifies individuals with PD at highest risk for rapid progression. The DM subtype presents with greater baseline motor and non-motor disease burden compared to other subtype classifications. Further, it exhibited a 50% milestone attainment rate within 10 years duration, significantly outpacing both traditional clinical classifications (TD, PIGD, indeterminate) and pathological subtypes (brain-first, body-first). Interestingly, DM patients showed accelerated decline across multiple domains, including cognition, motor complications, and functional dependence, with competing risks regression confirming a hazard ratio of 2.02 compared to the MMP subtype. In contrast, clinical subtyping offered limited prognostic value, as differences between TD, PIGD, and indeterminate groups were modest and often non-significant. This aligns with prior work by von Coelln *et al*.[23], who demonstrated that motor-based subtypes lack temporal stability and exhibit poor reproducibility across timepoints, limiting their use in longitudinal studies and clinical trials. Similarly, while body-first pathological subtypes progressed faster than brain-first, their predictive utility was less than the data-driven subtypes. CSF pTau and tTau concentrations differed significantly among clinical subtypes but the significance of these findings (P = 0.01 and P = 0.03, respectively) may not survive correction for multiple comparisons and should therefore be interpreted with caution.

Although non-specific, NfL has emerged as a consistent biomarker of axonal neurodegeneration and poor prognosis in PD. Multiple studies have demonstrated that elevated NfL levels correlate with faster motor and cognitive decline, shorter survival, and more severe clinical progression, especially in atypical or aggressive PD phenotypes [24–26]. In our study, serum NfL levels were significantly elevated in the DM subtype compared to MMP and IM subtypes (P = 0.005, post-hoc analysis), suggesting a greater burden of neurodegeneration. Further, we found that out of the examined biomarkers, serum NfL was a significant independent predictor of faster milestone attainment (*data not shown*), reinforcing its prognostic relevance. These findings support the emerging interpretation that NfL elevation may reflect an active axonal injury process that underpins the aggressive disease trajectory in the DM group. This aligns with prior findings by Pilotto *et al*., who identified that high NfL levels were associated with a more malignant PD phenotype and rapid motor progression over two years [24].

Our findings align with and extend prior work by Fereshtehnejad *et al*.[14, 27, 28], who first proposed the data-driven DM, IM, and MMP subtypes. This prospective validation in the PPMI cohort strengthens the clinical relevance of the data-driven subtyping framework. Further supporting the clinical significance of the DM subtype, Johansson *et al*.[29] analyzed the two-year longitudinal data demonstrating that patients classified under the DM subtype exhibited significantly faster progression in both motor and non-motor symptoms compared to other subtypes. Their findings highlight the aggressive nature of the DM phenotype and underscore its potential as a critical target for strati ed therapeutic interventions. In parallel, Hähnel *et al*.[30] proposed another data-driven progression model using multi-cohort clustering of longitudinal symptom data. Their analysis identified distinct fast- and slow-progressing PD subtypes which may parallel the DM vs. MMP subtypes in Fereshtehnejad model [14, 27, 28], a major shortcoming of their work is the lack of recommended or operational criteria to classify individual patients prospectively. This limits the clinical and translational applicability of their model.

On the other hand, our results challenge assumptions about pathological subtypes. The brain-first/body-first framework, based on Braak’s staging hypothesis [31] suggests that body-first patients exhibit faster progression due to widespread synucleinopathy [16]. This is consistent with our results that body-first patients progressed faster than brain-first, however, their progression rates remained inferior to DM, suggesting that pathological classifications may lack the granularity needed for trial strati cation. A prior study by Xu *et al*.[19] found faster disease progression in body-first PD compared to brain-first PD, however, they examined disease progression by the changes in MDS-UPDRS scores only [19]. In contrast, our study employed the 25 previously validated PPMI progression milestones [21]. The superiority of the DM subtype in predicting disease-progression may arise from the data-driven subtyping criteria itself. Since DM subtype is defined based on the scores of RBDSQ, MoCA, SCOPA-AUT, and MDS-UPDRS, it has more potential to capture individuals with impairment in multiple domains. However, in our study and others’ [19], the RBDSQ was used to de ne body-first/brain-first subtypes, which may not fully capture underlying neuropathology.

These findings have substantial implications for designing future disease-modification trials. Our simulations revealed that trials enrolling DM patients could achieve 80% statistical power with substantially smaller sample sizes (~ 50% fewer participants) and shorter durations compared to unstratified cohorts. In other words, for disease-modification trials aiming to slow the disease progression, prospective subtyping PD patients and enrollment of DM subtype will achieve greater statistical power and a higher likelihood of demonstrating treatment efficacy even with the same sample size and follow-up duration used in conventional trials of unstratified PD. This may be explained by the DM’s rapid progression, which enhances the sensitivity to detect treatment effects. Such strati cation could lower trial costs, accelerate timelines, and minimize participant exposure to ineffective therapies. Furthermore, focusing on rapid progressors aligns with ethical obligations to prioritize patients most likely to bene t from early intervention. These advantages position data-driven subtyping as a promising strategy for overcoming historical challenges in PD disease-modification trials, where high heterogeneity has contributed to failures.

Currently, the neurobiological basis of PD subtypes remains unknown. For future studies, one interesting question is whether those patients with DM subtype have a higher burden of α-synuclein pathology and/or more aggressive microglial activation that contributes to the faster disease progression and the active neurodegenerative process (as reflected by the higher CSF and serum NfL levels). A recently made available preprint reported that Amplification parameters of the alpha-synuclein seed amplification assay on CSF were distinct between PD data-driven subtypes, suggesting that patients with more aggressive disease (those subtyped as DM) have shorter reaction time, which may reflect higher density of misfolded α-synuclein aggregates in the CSF sample [32]. Future advances in α-synuclein quantification methods and neuroimaging modalities would help better understanding of disease behavior in those patients. Lack of subtype stability has been criticized in the clinical subtyping model (TD, PIGD, and indeterminate), because disease subtypes may reflect different stages of the disease rather than distinct clinicopathological entities [23]. For this reason, future longitudinal studies tracking subtype stability over time are also needed, as PD heterogeneity may evolve with disease course. Finally, interventional trials should test whether subtype-specific therapies (e.g., targeting neuroinflammation in DM) yield superior outcomes compared to the “one-size-fits-all” approaches.

This study has several strengths: (1) the use of PPMI, a well-characterized large cohort of PD patients, (2) rigorous evaluation of disease progression across six functional domains instead of focusing on motor score only, (3) the integration of competing risks regression and disease-modification trial size estimations further strengthen the clinical applicability of our findings. However, several limitations warrant consideration. First, the PPMI cohort comprises early-stage PD patients (Hoehn & Yahr I–II) without dementia, limiting generalizability to more advanced stages or those with significant cognitive impairment. Second, while milestone definitions were clinically meaningful, they may not fully reflect the biological process underlying the disease progression. Third, survival bias may exist if patients who dropped have different progression rates. We handled missing milestone assessments conservatively by assuming that if data were missing, the milestone was not yet reached. Finally, the single-cohort design requires replication in other independent populations, particularly those with diverse demographics and longer follow-up.

## Conclusions

In conclusion, data-driven subtyping, particularly the identification of the DM subtype, could optimize disease-modification clinical trials for PD. Given the rapid disease progression observed in this subtype, this approach is expected to increase the sensitivity of disease-modification trials to demonstrate benefits by enhancing statistical power, reducing trial costs, and accelerating the evaluation of disease-modifying therapies. As PD research advances toward precision medicine, the integration of multimodal biomarkers into subtyping algorithms will be crucial for elucidating the neurobiological basis of PD heterogeneity.

## Figures and Tables

**Table 1:** Baseline Clinical Characteristics Across Parkinson’s Disease Subtypes

Clinical Subtypes					
	N	TD (n = 958)	PIGD (n = 165)	Indeterminate (n = 97)	P value
Age at enrollment	1192	63.8 (56.6–70.0)	64.6 (56.2–69.9)	63.1 (55.6–73.0)	P = 0.70
Age at symptom onset	1209	61.6 (54.6–67.8)	62.7 (53.8–68.4)	61.1 (52.7–69.6)	P = 0.66
Age at diagnosis	1220	63.1 (55.8–69.2)	64.2 (55.8–69.4)	61.8 (54.5–71.6)	P = 0.58
Sex: male	1220	626/958 (0.7)	106/165 (0.6)	42/67 (0.6)	P = 0.95
MDS-UPDRS I	1203	5.0 (3.0–8.0)	7.0 (3.7–10.3)	6.0 (3.9–10.1)	P < 0.01*
MDS-UPDRS II	1215	5.0 (3.0–8.0)	8.0 (4.0–12.0)	7.0 (4.0–12.0)	P < 0.01*
MDS-UPDRS III (off)	1184	21.0 (15.0–28.0)	20.0 (14.0–28.0)	23.0 (17.0–33.8)	P = 0.88
MDS-UPDRS total	1171	32.0 (24.0–42.0)	38.0 (24.0–48.0)	40.0 (28.0–50.1)	P = 0.01*
UPSIT total	451	22.0 (15.0–28.0)	22.0 (14.7–28.0)	19.0 (13.3–27.0)	P = 0.89
MoCA total	1214	27.0 (25.0–29.0)	27.0 (25.0–29.0)	27.0 (25.9–28.0)	P = 0.98
RBDSQ total	1215	3.0 (2.0–5.0)	3.0 (2.0–6.3)	4.0 (2.0–5.8)	P = 0.10
SCOPA-AUT total	1210	9.0 (6.0–13.0)	10.0 (6.0–14.0)	11.0 (6.0–15.8)	P = 0.30

Pathological Subtypes				
	N	Brain-first (n = 646)	Body-first (n = 319)	P value
Age at enrollment	1192	63.1 (55.8–69.8)	65.1 (59.1–70.4)	P = 0.01*
Age at symptom onset	1209	61.1 (53.7–67.7)	63.2 (55.8–68.4)	P = 0.03*
Age at diagnosis	1220	62.6 (55.2–69.0)	64.4 (58.0–69.7)	P = 0.02*
Sex: male	1220	418/646 (0.6)	234/319 (0.7)	P = 0.01*
MDS-UPDRS I	1203	4.0 (2.0–7.0)	7.0 (5.0–11.0)	P < 0.01*
MDS-UPDRS II	1215	4.0 (2.0–7.0)	7.0 (4.0–12.0)	P < 0.01*
MDS-UPDRS III (off)	1184	20.0 (15.0–27.0)	21.0 (16.0–30.0)	P = 0.03*
MDS-UPDRS total	1171	30.0 (23.0–39.0)	37.0 (27.0–49.3)	P < 0.01*
UPSIT total	451	23.0 (16.0–28.0)	19.0 (13.2–26.8)	P = 0.02*
MoCA total	1214	27.0 (25.0–29.0)	27.0 (26.0–28.0)	P = 0.06*
RBDSQ total	1215	2.0 (1.0–3.0)	8.0 (7.0–10.0)	P < 0.01*
SCOPA-AUT total	1210	8.0 (5.0–11.0)	12.0 (8.0–18.0)	P < 0.01*

Data-driven Subtypes					
	N	MMP (n = 422)	IM (n = 536)	DM (n = 232)	P value
Age at enrollment	1192	60.8 (53.6–66.7)	65.2 (58.4–70.3)	66.7 (61.0–72.9)	P < 0.01*
Age at symptom onset	1209	58.8 (51.2–64.6)	63.1 (56.0–68.5)	64.5 (58.5–70.5)	P < 0.01*
Age at diagnosis	1220	60.5 (53.1–66.0)	64.5 (57.0–69.5)	65.7 (60.4–72.4)	P < 0.01*
Sex: male	1220	267/422 (0.6)	349/536 (0.7)	158/232 (0.7)	P = 0.46
MDS-UPDRS I	1203	4.0 (2.0–6.3)	6.0 (3.0–8.0)	9.0 (6.0–13.0)	P < 0.01*
MDS-UPDRS II	1215	4.0 (2.0–6.0)	5.0 (3.0–8.0)	11.0 (7.0–14.0)	P < 0.01*
MDS-UPDRS III (off)	1184	18.0 (13.0–23.0)	20.0 (15.0–26.0)	33.5 (27.0–39.0)	P < 0.01*
MDS-UPDRS total	1171	27.0 (20.0–33.0)	33.0 (24.0–40.0)	52.0 (45.0–60.0)	P < 0.01*
UPSIT total	451	24.0 (17.0–29.0)	22.0 (15.0–28.0)	19.0 (13.0–26.6)	P = 0.01*
Clinical Subtypes				
	N	TD (n = 958)	PIGD (n = 165)	Indeterminate (n = 97)	P value
MoCA total	1214	28.0 (27.0–29.0)	27.0 (25.0–28.0)	26.0 (24.0–28.0)	P < 0.01*
RBDSQ total	1215	2.0 (1.0–3.0)	4.0 (2.0–7.0)	6.0 (3.0–8.0)	P < 0.01*
SCOPA-AUT total	1210	7.0 (4.0–10.0)	10.0 (6.0–14.0)	15.0 (10.0–19.6)	P < 0.01*

Values are presented as median (interquartile range) unless otherwise indicated; N is the number of non-missing values; P-values are based on Wilcoxon test for comparisons of 2 subtypes (pathological subtypes), Kruskal-Wallis test for continuous variables compared across 3 subtypes (clinical and data-driven subtypes), and Pearson chi-square test for categorical variables

* statistically significant at the level of 0.05.

**Table 2:** CSF and Serum Biomarker Levels Across Subtypes

Clinical Subtypes					
	N	TD (n = 958)	PIGD (n = 165)	Indeterminate (n = 97)	P value
CSF Aβ42	443	844.3 (613.7–1126.0)	874.0 (637.1–1121.8)	745.5 (558.6–1023.2)	P = 0.32
CSF pTau	491	13.0 (10.2–16.8)	14.4 (11.3–19.1)	11.8 (9.2–13.4)	P = 0.01*
CSF tTau	491	153.8 (122.5–199.5)	172.2 (139.8–214.9)	143.7 (120.4–164.2)	P = 0.03*
CSF NfL	212	92.9 (68.1–122.8)	82.4 (56.3–115.7)	74.8 (62.1–103.3)	P = 0.29
Serum NfL	453	11.8 (8.4–16.0)	11.6 (8.5–15.3)	11.9 (8.7–18.7)	P = 0.82

Pathological Subtypes				
	N	Brain-first (n = 646)	Body-first (n = 319)	P value
CSF Aβ42	443	828.2 (628.5–1068.9)	874.1 (588.8–1206.1)	P = 0.69
CSF pTau	491	12.8 (10.4–16.7)	13.5 (9.7–17.7)	P = 0.56
CSF tTau	491	152.8 (123.3–195.0)	167.1 (120.9–213.5)	P = 0.17
CSF NfL	212	83.7 (64.6–117.1)	95.1 (67.9–119.3)	P = 0.34
Serum NfL	453	11.7 (8.2–16.1)	11.8 (8.5–16.4)	P = 0.69

Data-driven Subtypes					
	N	MMP (n = 422)	IM (n = 536)	DM (n = 232)	P value
CSF Aβ42	443	842.2 (646.0–1197.0)	847.0 (610.3–1079.6)	843.6 (548.1–1075.3)	P = 0.64
CSF pTau	491	12.6 (10.1–16.7)	13.0 (10.5–17.5)	13.7 (10.1–17.4)	P = 0.35
CSF tTau	491	151.6 (120.3–196.9)	155.5 (125.1–202.7)	166.6 (128.8–206.6)	P = 0.30
CSF NfL	212	74.8 (58.0–110.3)	94.1 (70.6–118.3)	104.0 (72.6–136.2)	P < 0.01*
Serum NfL	453	10.3 (7.5–14.6)	12.3 (8.9–16.9)	14.3 (10.0–18.3)	P < 0.01*

CSF Aβ42: cerebrospinal fluid amyloid-beta 42; CSF pTau: cerebrospinal fluid phosphorylated tau; CSF tTau: cerebrospinal fluid total tau; CSF NfL: cerebrospinal fluid neuro filament light chain; Serum NfL: serum neurofilament light chain. Values are presented as median (interquartile range); N is the number of non-missing values; P-values are based on Wilcoxon test for comparisons of 2 subtypes (pathological subtypes) and Kruskal-Wallis test for continuous variables compared across 3 subtypes (clinical and data-driven subtypes)

* statistically significant at the level of 0.05.

**Table 3:** Competing Risks Regression for Progression Milestone Attainment Across Subtypes

Subtype Comparison	SHR†	95% Confidence Interval	P value
Clinical Subtypes			
PIGD vs TD	1.34	(1.00 to 1.79)	0.043
Indeterminate vs TD	1.36	(0.96 to 1.93)	0.079
Pathological Subtypes			
Body-First vs Brain-First	1.40	(1.09 to 1.79)	0.007
Data-Driven Subtypes			
DM vs MMP	2.02	(1.49 to 2.75)	< 0.001
IM vs MMP	1.53	(1.19 to 1.95)	0.001
Enrollment age covariate			
Clinical subtypes model	1.02	(1.00 to 1.02)	0.006
Pathological subtypes model	1.01	(0.99 to 1.02)	0.056
Data-driven subtypes model	1.00	(0.99 to 1.02)	0.165

† TD = Tremor dominant; PIGD = Postural Instability Gait Disorder; MMP = Mild Motor Predominant; IM = Intermediate; DM = Diffuse Malignant; Hazard ratios are based on Fine-Gray competing risks regression; models are adjusted for age at enrollment.

## Data Availability

The datasets analyzed during the current study are publicly available from the Michael J. Fox’s PPMI database: http://PPMI-info.org. The statistical analysis files: Stata script files (.do), Jamovi files (.omv), and the final dataset (.csv) are available from the corresponding author on reasonable request.
